# A Facile Approach for the Mass Production of Submicro/Micro Poly (Lactic Acid) Fibrous Mats and Their Cytotoxicity Test towards Neural Stem Cells

**DOI:** 10.1155/2016/8921316

**Published:** 2016-09-06

**Authors:** Afra Hadjizadeh, Houman Savoji, Abdellah Ajji

**Affiliations:** ^1^Department of Biomedical Engineering, Amirkabir University of Technology, Tehran 1591634311, Iran; ^2^CREPEC, Department of Chemical Engineering, École Polytechnique de Montréal, Montreal, QC, Canada H3C 3A7; ^3^Industrial Materials Institute, National Research Council Canada, Boucherville, QC, Canada J4B 6Y4; ^4^Institute of Biomedical Engineering, École Polytechnique de Montréal, Montreal, QC, Canada H3C 3A7

## Abstract

Despite many of the studies being conducted, the electrospinning of poly (lactic acid) (PLA), dissolved in its common solvents, is difficult to be continuously processed for mass production. This is due to the polymer solution droplet drying. Besides, the poor stretching capability of the polymer solution limits the production of small diameter fibers. To address these issues, we have examined the two following objectives: first, using an appropriate solvent system for the mass production of fibrous mats with fine-tunable fiber diameters; second, nontoxicity of the mats towards Neural Stem Cell (NSC). To this aim, TFA (trifluoroacetic acid) was used as a cosolvent, in a mixture with DCM (dichloromethane), and the solution viscosity, surface tension, electrical conductivity, and the continuity of the electrospinning process were compared with the solutions prepared with common single solvents. The binary solvent facilitated PLA electrospinning, resulting in a long lasting, stable electrospinning condition, due to the low surface tension and high conductivity of the binary-solvent system. The fiber diameter was tailored from nano to micro by varying effective parameters and examined by scanning electron microscopy (SEM) and image-processing software. Laminin-coated electrospun mats supported NSC expansion and spreading, as examined using AlamarBlue assay and fluorescent microscopy, respectively.

## 1. Introduction

Porous polymeric structures with high porosity and interconnected pores have potential use in many biomedical applications [1–3] like protective fabrics, wound dressing [4] drug delivery [5–7], tissue engineering scaffolds [8, 9], and so on [10]. This is due to their highly porous microstructure with interconnected pores and large surface area. Among the various techniques developed for producing potential biomaterials for tissue engineering [11–15], electrospinning, being a simple, low-cost, and a potent method for manufacturing porous structures nanoscale polymer fibers, is the most suitable and versatile technique for the tissue engineering applications. This technique has the capability of producing nano/microfibrous scaffolds [16] using various natural macromolecules, synthetic polymers [17], and their mixtures to mimic the structure and the function of a native extracellular matrix (ECM) [18].

Electrospinning relies on the induction of electrical charges within a spinnable polymer fluid by applying a high voltage to the fluid. When the fluid gains enough charges, called the critical charge amount, a fluid jet will start to erupt from the droplet formed at the tip of the conductive needle, leading to the formation of a cone shape called the Taylor cone. The jet will fly towards a grounded collector that is the region of negative potential. The parameters that affect electrospinning, and as a result of the fiber properties, can be classified into the polymer solution properties, process parameters, and ambient conditions. The process parameters include the applied voltage, flow rate, tip to collector distance, and collector geometry. By understanding these parameters and changing them, one can produce fibrous structures with various morphological, physical, and mechanical properties [19].

One of the polymers that have been long used in biomedical applications is PLA. In recent years, the need for fibrous PLA structures has increased significantly and many protocols to produce it by the electrospinning technique have been reported [20–22]. Several solvents for PLA exist that can produce nanofibers by electrospinning. For instance, fluorine-based solvents, such as hexafluoro-2-propanol, are used for fiber production in small quantities in the laboratory [23]. However, these solvents are expensive. The less expensive solvents which are commonly used to prepare electrospinning solutions are chloroform (CHCL_3_), dichloromethane (CH_2_CL_2_, DCM), dimethyl formamide (DMF), and their mixtures. However, it is very difficult to establish a stable electrospinning condition for the mass production of uniform PLA fibers which needs a long lasting electrospinning process. A major problem with using these solvents is the drying of polymer solution droplets on the tip of the needle (nozzle). Using low polymer concentrations could be a solution, but it leads to bead formations [24]. To overcome this issue, the conductivity of the spinning solution should be increased, leading to its high stretchability and as a result the production of ultrafine fibers. This can be achieved by adding an organic or inorganic ionizable substance, such as NaCl. However, this method also suffers from some drawbacks, such as undesirable interactions with other solutes or solvents.

The conductivity of the solution can be also enhanced by the solvent type. For example, the conductivity of DMF (6 × 10^−2^ *μ*s/cm^−1^, 1 siemen(s) = 1 mho) is higher than that of DCM (4 × 10^−5^ *μ*s/cm^−1^). Thus, DMF can be used in a mixture with DCM to increase the conductivity of the solution [19]. A very simple method to address the issues of droplet coagulation and low stretching could be to involve a less volatile and highly conductive solvent, such as TFA (trifluoroacetic acid) (bp: 72.4°C). Therefore, in this study, TFA has been mixed as a cosolvent with DCM, to produce PLA nano- and microfibers towards a stable electrospinning process. We have tested commonly used single and binary-solvent systems, including chloroform, DCM, and DCM/DMF, and compared them with DCM/TFA, in order to find a suitable solvent system for the mass production of PLA nano/microfibrous mats.

Neural Stem Cells (NSCs) have the potential of self-renewal, as well as differentiating into different neural cell types [25]. This offer advantages for NSC based cell therapies to treat neurodegenerative diseases and traumatic injuries. However, to apply this strategy in clinical applications, appropriate approaches are needed for large scale cell expansion. With regard to their nanofibrous microenvironment* in vivo*, with specific physical, biochemical, and topographical properties, electrospun fibrous mat could provide a similar artificial environment to support cell function and to achieve the above-mentioned goals. There are few published studies on the effect of electrospun structures towards NSC behavior [26–28], but a lot more remains to be learned. To this aim, we have further tested the prepared electrospun PLA mats towards NSC viability and expansion using AlamarBlue assay and fluorescent microscopy.

## 2. Methodology

PLA 3051D (Mn = 93,500 g/mol) was obtained from the Nature Works USA. Analytical grade solvents, from Sigma-Aldrich, were used as received to prepare electrospinning solutions.  Table 1 shows some properties of the selected solvents.

PLA was dissolved in various solvents such as chloroform, DCM, DMF, DCM/DMF, and DCM/TFA in various concentrations. The required amounts of the polymer in granule form were weighed and added to a glass 10 mL vial, then the required volume of solvent was added to prepare solutions in different concentrations (between 2 and 15% (w/v)), and the contents were stirred on a magnetic stirrer until the polymer was completely dissolved (for 24 hours), at room temperature. TFA containing samples were stirred only 4 hours, leading to a complete polymer dissolution. Then, the spinnability of the solutions was examined using a homemade electrospinning set-up. The stability of the Taylor cone and continuity of the jet projection toward collector were recorded by a camera set-up (including a lens and a computer system). The process was recorded from the start (second 1) to about 5 hours. PLA solutions in two different concentrations (7.7% (w/v) and 10% (w/v)) were chosen for further investigations at various flow rates ranging from 0.1 to 10 mL/hr. The obtained fibers were collected on an either stationary or rotating collector. The morphology and diameter of electrospun fibers were characterized using SEM and image-processing software (Image J).

### 2.1. Electrospinning

Submicro/microfibers were produced, using a homemade electrospinning set-up. The set-up had a high voltage power supply (Gamma Inc.), a syringe pump (PHD 4400, Harvard Apparatus) to deliver the polymer solution at given flow rates, and a stainless steel rotating drum collector (15 cm in diameter, 35 cm in length). Glass syringes (1 mL and 5 mL) connected to a stainless steel needle gauge (Popper & Sons Inc.) were used to deliver the polymer solution. The electrospinning conditions, used in this study, including polymer solution parameters, process parameters, and ambient parameters were summarized in Table 2.

### 2.2. Characterization of PLA Solutions

The rheological properties of the PLA solutions were measured with a vibrating Viscometer (VC-10 A&D Co., Japan) at a frequency of 30 Hz at room temperature (22°C). The surface tension of the PLA solutions was measured using a surface tension tensiometer (OCA20, Data Physics Instruments GmbH) at room temperature. The electrical conductivity of the polymer solutions was determined using a digital conductivity meter (2700 Series Benchtop Meters, Thermo Fisher Scientific, USA) that was calibrated with standard solutions. Each experiment was carried out in triplicate to ensure reproducibility and the average of the results was reported.

### 2.3. Taylor Cone and Jet Visualization

In order to have a reproducible electrospinning process with a continuously running stable jet, it is crucial for the maintaining of a well-balanced potential and flow rate during the electrospinning process. This can be achieved by the fine tuning of these process parameters using a camera system to visualize the tip of the needle delivering the polymer solution as well as the jet released from it. To this aim, we have used a homemade camera system equipped with a 100x lens and computer systems to observe the Taylor cone formation and the polymer solution jet projection continuity in using different solvent systems.

### 2.4. Effect of Process Parameters

Once a more suitable solvent system (TFA/DCM (1 : 1)) was found, the PLA solution concentrations of 7.7% and 10% (w/v) were chosen for further investigations. The concentration of 7.7% (w/v) exhibited the lower limit for producing smooth fibers and 10% (w/v) the higher limit. In the polymer solution concentration below 7.7% beads were observed on the fibers and above 10% the process did not run smoothly. The smallest fiber diameter was obtained at the lower polymer solution concentration, larger tip-collector distance, and lower flow rates. However, for larger fiber diameter, the process was performed at a high polymer solution concentration, high flow rate, and short tip-collector distance. The concentrations of 7.7% were spun with a tip-collector distance of 15 cm, flow rates ranging from 0.1 to 1 mL/hr and a voltage of 16–18 kV. The concentration of 10% (w/v) was processed using a tip-collector distance of 13.5 cm, a flow rate from 0.1 to 10 mL/hr, and a voltage of 13–30 kV, as listed in Table 2.

Two different collector speeds in both TD (transverse direction) (0.3 m/min and 1 m/min) and MD (machine direction) (2.25 m/s and 3.6 m/s) were utilized to produce the electrospun PLA nonwoven mats with a width of 5 cm and 11 cm, a length of 47 cm, and a thickness of 50–60 *μ*m. The mats, produced using 7.7% and 10% (w/v) in different collector speed, were named (A1-A2) and (B1-B2), respectively, as indicated in Table 2.

### 2.5. PLA Fiber Diameter Measurement

The samples were gold-platinum coated for 2 min, after mounting on the SEM holders. Imaging was performed using a SEM (JEOL, JSM-6100) (for samples from at least two experiments with duplicate samples) at a voltage of 15 kV, and images were obtained at different magnifications. Using an image-processing software (Image J, Scion Co., Frederick, Maryland), the diameters of at least 100 fibers were randomly measured for each condition, on the obtained SEM images. The results of fiber diameter measurement were reported in Table 3.

### 2.6. Porosity Measurement

The samples were freely placed on a flat surface and then were gently flattened. A digital caliper was used to measure the width and length of the samples. The mat thickness was measured using a digital thickness gauge (Film Master, Qualitest), designed for film thickness measurement, with a 0.001 mm resolution. The following equation (1) [16] was used to measure the porosity:

(1) $$\begin{array}{c} Porosity\left(\;\%\;\right)=\left(\;1-\frac{ad}{bd}\;\right)\times 100, \end{array}$$

where the ad is PLA apparent density (g/cm^3^) (i.e., PLA mat mass (g)/PLA mat thickness (cm) × PLA mat area (cm^2^)) and bd is the bulk density of pure amorphous PLA (1.25 gr*/*cm^3^). This measurement was performed for at least two experiments with duplicate samples. The obtained results were shown in Table 3.

### 2.7. Biological Experiment

PLA mats with fiber diameters of 0.4, named NF, were used to investigate the NSCs expansion. The mats were cut in a circular shape, placed in 24 well plates and sterilized with ethanol (70%). Then they were immersed in a laminin solution (10 *μ*g/mL in PBS (+Ca/+Mg)) for 1 hr at room temperature.

The samples were rinsed with PBS and seeded with NSC (Lonza, USA) (25000 cells/well) in complete culture medium (neural progenitor maintenance medium (NPMM)) (Catalog number CC-3209 Lonza, USA) and incubated in a CO_2_ incubator at 37°C and 5% CO_2_. Cell viability and proliferation were evaluated by using AlamarBlue (Invitrogen, cat.# DAL1100) and observed for 14 days. The cells were stained with Phalloidin-TRITC (1 : 100 dilution, cat. P1951, Sigma Chemical Co.) for actin filament and SYTOX Green Nucleic Acid Stain (1 *μ*M, cat. S7020, Molecular Probes, Eugene, OR) in the nucleus, and their morphology was evaluated using fluorescence microscopy (Axio observer Z1, Zeiss).

### 2.8. Statistical Analysis

The statistical analysis of the data was performed using statistical package for the social sciences (SPSS, SPSS Inc.) software. For the data, analysis of variance (ANOVA) was used. Results were expressed as mean ± standard deviation. The significance of differences at 0.05 levels was evaluated.

## 3. Result and Discussion

**Figure 1:**
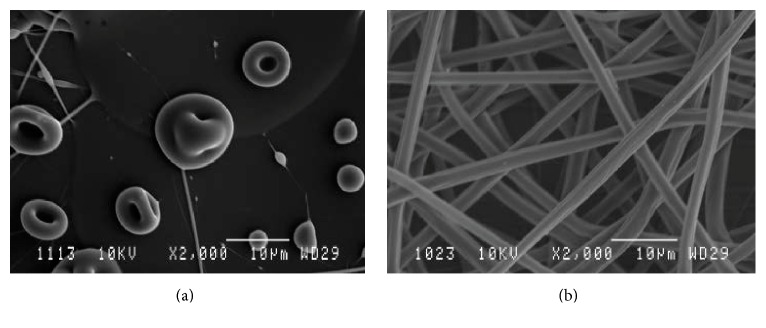
Representative images of PLA electrospinning: (a) solution concentration 2.7% (w/v) in chloroform; (b) solution concentration 7% (w/v) in chloroform.

**Figure 2:**
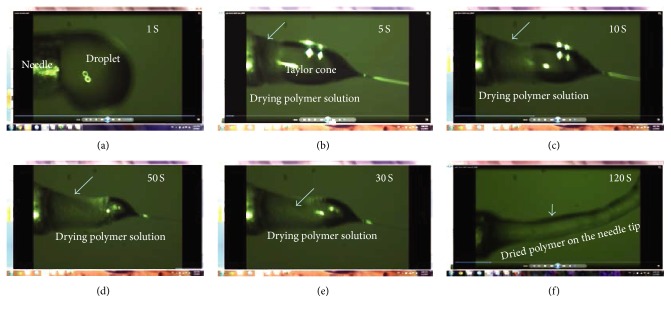
Representative images showing PLA solution droplet coagulation on the needle tip when PLA is dissolved in dichloromethane: (a–f) periodic snapshots from needle tip during the electrospinning process.

PLA dissolved in chloroform, DCM, DMF, and their mixtures in low concentration (2.7–3% (w/v)) produced droplets (Figure 1(a)) in the case of pure chloroform and beaded fibers with the rest of the above-mentioned solvent systems. By increasing polymer solution concentration to 7% (w/v), bead fewer fibers were obtained (Figure 1(b)). However, polymer solutions, prepared using chloroform, DCM, DMF, and their mixtures, dried on the needle tip, a few seconds after, hindering the continuous fiber production. Therefore, the process did not last longer than 1-2 minutes, as recorded by a camera system (Figure 2).

**Figure 3:**
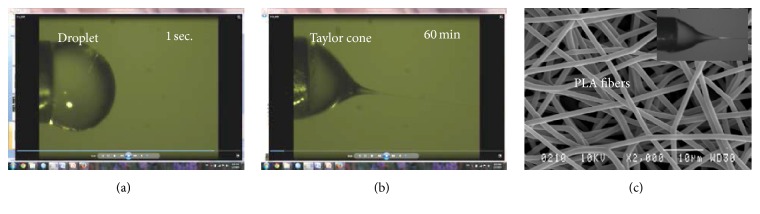
Representative images showing PLA electrospinning process with stable and nondrying Taylor cone and smooth fiber production. PLA is dissolved in dichloromethane and trifluoroacetic acid mixture (1 : 1).

**Figure 4:**
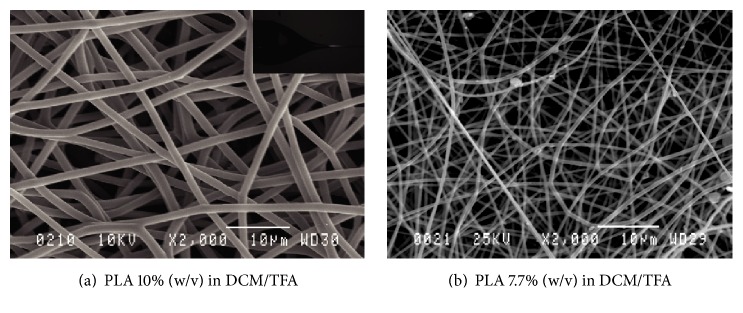
Representative SEM images of electrospun PLA, obtained in two different polymer solution concentrations: 10% (a) and 7.7% (b).

**Figure 5:**
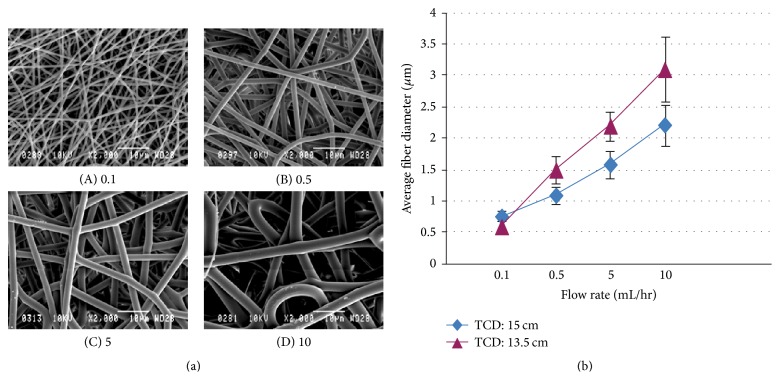
(a) Representative SEM images of electrospun PLA (10% (w/v)) with various fiber diameters, obtained in various polymer solution flow rates (0.1–10 mL/hr). (b) The effect of PLA (10% (w/v)) solution flow rate on fiber diameter in two different tip-collector distances (TCD). *N* > 3; *N* is the number of analyses.

On the other hand, when a mixture of DCM/TFA was used, a stable Taylor cone (Figure 3) and a long lasting electrospinning process with different PLA concentrations were observed. Hence, by using a solvent mixture of DCM/TFA, it was practical to produce PLA mats in large amounts. By varying process parameters, such as polymer solution concentration and flow rate, it was possible to obtain PLA mats with various fiber diameters, as shown in Figures 4 and 5.

### 3.1. Taylor Cone and Jet Stability

For the electrospinning of polymers, volatile solvents are often used to dissolve the polymer. This is to ensure that most of the solvent will evaporate leading to the formation of individual fibers during the flight of electrospinning jet towards the collection plate. However, solvent evaporation may cause clogging of the polymer during the stretching of the solution from the needle, preventing a stable Tylor cone and as a result jet formation. Therefore, effective methods are needed to be used in order to maintain jet stability during the process. To achieve this goal, it is crucial to monitor the process in order to maintain a balance between the desired electric potential and flow rate during the initial stages of the electrospinning process. Using a camera system, the tip of the needle delivering the polymer solution and the jet releasing from the nozzle was visualized during the process. As shown in Figure 2, it was observed that PLA solutions prepared using common solvents such as chloroform, DCM, DMF, and DCM/DMF mixtures started to dry and block the nozzle immediately about 1 second after coming out of the needle and ending up with a completely dried polymer on the needle tip in a few minutes. Thus, fiber collection was impossible or the tip had to be regularly cleaned. However, in using DCM/TFA mixture as the solvent, a very stable Taylor cone, and a stable polymer solution jet projection towards a collector was observed (Figure 3) during many hours of the process.

### 3.2. Effect of Solvent and Polymer Solution Properties

Electrospinning requires just the simple equipment to produce fibrous mats with nano- to microfibers. However, with all the apparent simplicity, it has a complicated science behind. These include the electrostatic force analysis, fluid rheology, and polymer solution properties. In the case of polymer solutions, properties such as solvent evaporation rate, surface tension, and solution conductivity affect jet stability, fiber structure, and properties.

Surface tension in a liquid surface is an intrinsic inward contractive force by which the fluid resists an external force. The surface tension is originated by the cohesive forces between liquid molecules. Electrospinning is initiated when the electrostatic force of the charged solution overcomes its surface tension. Surface tension decreases the surface area per unit mass of a fluid. Therefore, for a high concentration of free solvent molecules, surface tension makes the solvent molecules take a spherical shape. Greater interaction occurs between the solvent and polymer molecules at a higher viscosity. Thus, the charges make the solution be stretched, causing the solvent molecules to spread over the entangled polymer molecules. This will reduce the tendency for the solvent molecules to come together under the influence of surface tension. Surfactant or solvents having low surface tension, such as ethanol, can be added to reduce surface tension and will result in the formation of more uniform and smooth fibers. By the acceleration of the electrospinning jet towards the collector, the solvent evaporates, resulting in dry individual fiber formation and deposition on the collector. The solvent evaporation rate is influenced by various factors such as vapor pressure, boiling point, specific heat, enthalpy and heat of vaporization of the solvent, the rate of heat supply, solvent-solvent and solvent-solute molecule interaction, the surface tension of the liquid, and ambient condition.

Low concentration PLA solution (3%) in all solvent did not produce fibers due to their high surface tension (Table 4). However, PLA dissolved in DCM + TFA produced stable Taylor cone and smooth fibers which confirm the effect of low surface tension on electrospinability of the polymer solution (Table 4).

The ability of an electrolyte solution to conduct electricity is called conductivity (or specific conductance). The ability of the solution to carry charges determines the initiation and subsequent stretching of the electrospinning jet toward the collector. Therefore, sufficient charges are needed in the electrospinning solution to initiate the process, by generating repulsive forces to overcome the surface tension of the solution. Generally, solvents contain very few free ions, which are responsible for the electrical conductivity of solutions; thus they exhibit very low electrical conductivity. It is possible to increase the electrical conductivity of the solvent by adding acids and bases such as mineral acids, carboxylic acids, mineral salts, and dissolved carbon dioxide. This will facilitate the stretching of the jet, thus leading to the formation of the fibers with smaller diameters. However, the reduction in the fiber diameter is limited by the viscoelastic forces acting against the columb forces of the charges; the greater is the stretching the higher are the viscoelastic forces. Since the presence of ions increases the conductivity of the solution, the critical voltage for electrospinning to proceed is also reduced.

Facilitated electrospinning of PLA using TFA may be attributed to the fact that this substance increases the solution conductivity (Table 4). Changing the ion concentration, including H^+^ (presented as pH) of the solution, is a way to change its conductivity. By the use of TFA as an acid, the ions increase in the solution which increases the conductivity of the system. Since the higher conductivity of the solution provides higher electrostatic forces, the solution containing more ions can be stretched easily.  Table 1 shows the conductivity of the solvents used in this study. Fluorine-based solvents, such as hexafluoro-2-propanol, are also used for fiber production in small quantities in the laboratory [23]. However, these solvents are more expensive (at least three times) than TFA. Our results are in agreement with previously published studies. In a study, poly lactic acid (PLA) solutions have been prepared in various pure solvents and binary-solvent systems to produce nanofibers with different morphology and diameter [15]. In another study beaded fine fibers have been observed in low PLA concentrations in dichloromethane solution. The addition of pyridinium formiate (PF) has increased the electrical conductivity resulting in a significant reduction of bead formation [14]. However, none of these two studies have discussed the long lasting process for mass production of electrospun fibers.

### 3.3. Effect of Process Parameters

It was attempted to produce suitable PLA electrospun mats with determined fiber diameter, the smallest and largest possible fiber diameters only by varying the polymer solution concentration. This was done by varying the flow rate, collector-tip distance, and collector speed and adjusting the other process parameters accordingly. The three most effective parameters in fiber diameter size were the polymer solution concentration, flow rate, and tip-collector distance, as confirmed by the fiber morphology analysis. Depending on the polymer solution concentration, process parameters were adjusted and a stable Taylor cone was achieved. Then the electrospun fibers were deposited onto a stainless steel drum collector. It was observed that the fiber diameter was influenced by polymer solution concentration that played the first role, flow rate the second, and tip-collector distance the third. Usually, higher polymer solution concentration in the same solvent results in the higher viscosity and elasticity of the solution. This hinders stretching of the polymer solution jet, resulting in a larger fiber diameter. Moreover, by increasing the solution flow rate, the available polymer for electrospinning increases, leading to an increase in fiber diameter. When the tip-collector distance is short, the flight time for the jet decreases, which results in an increase in fiber diameter.

A higher polymer solution concentration (10% (w/v)) and a flow rate (10 mL/hr) produced a mat with a larger fiber diameter (3 *μ*m). In contrast, a fiber diameter of 600 nm was obtained using a polymer solution concentration of 7.7% (w/v) and flow rate of 0.5 mL/hr (Table 3 and Figure 4). Therefore, a fiber diameter in the range of submicron was obtained for further characterizations in the solution concentration of 7.7% (w/v), flow rate of 0.5 mL/hr, and tip-collector distance of 15 cm, whereas microfibers were produced in a 10% (w/v) polymer solution concentration, flow rate of 10 mL/h, and tip-collector distance of 13.5 cm.

The effects of polymer solution characteristics, process parameters (such as voltage, flow rate, and tip-collector distance), and collector motion on the properties of electrospun mats have been studied for polyethylene terephthalate (PET) submicro/microelectrospun fibers in our previous study [8, 16, 17]. The important role of some of them, including polymer solution concentration, flow rate, and collector motion on the fiber diameter and morphology, has been well established [8, 16, 17]. In this study, it was attempted to apply those findings to produce suitable PLA electrospun mats with determined fiber diameter.

**Figure 6:**
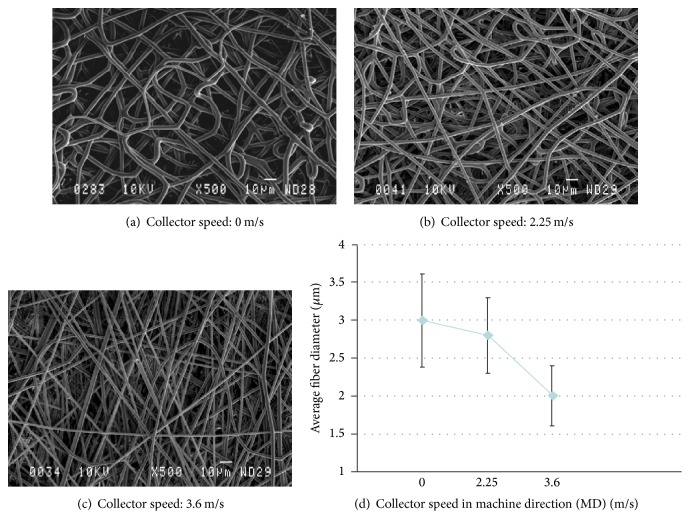
Representative SEM images of electrospun PLA (10% (w/v)), obtained in various rotating drum speeds in the machine direction (MD); transverse direction (TD) is constant at 0.3 m/min, *N* > 3; *N* is the number of analyses.

In addition to the polymer solution concentration and flow rate (Figures 4 and 5), collector speed also changed fiber diameter, as shown in Table 3 and Figure 6. This was examined for two different fiber diameters, produced by varying process parameters. The fiber diameter decreased when collector speed increased. This decrease was significant (^*∗*^ *P* < 0.05) (Table 3).

### 3.4. Cell Adhesion and Proliferation

**Figure 7:**
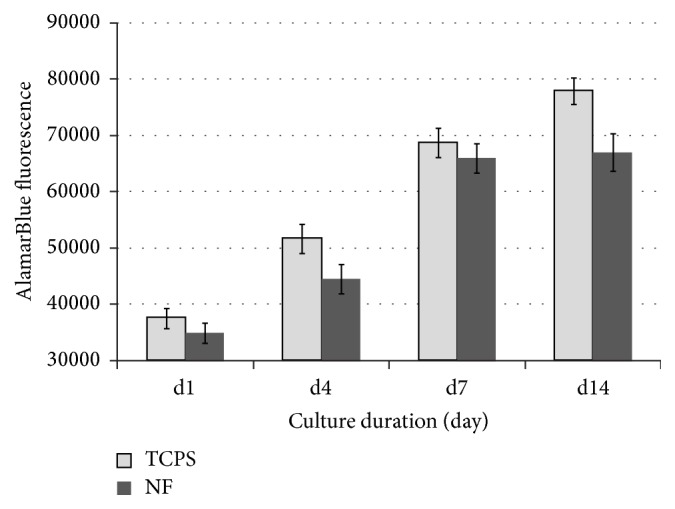
Cell viability as a function of the culture time measured by AlamarBlue assay. Cell proliferation on tissue culture polystyrene (TCPS) and submicrio-fiber (NF, 0.6 *μ*m) were presented.

Cell viability as a function of the culture time measured by the AlamarBlue assay on tissue culture polystyrene (TCPS) and submicrofiber (0.6 *μ*m) has been presented in Figure 7. The electrospun PLA mat supported cell adhesion and expansion with prolonged culture duration up to 14 days in the complete medium.

**Figure 8:**
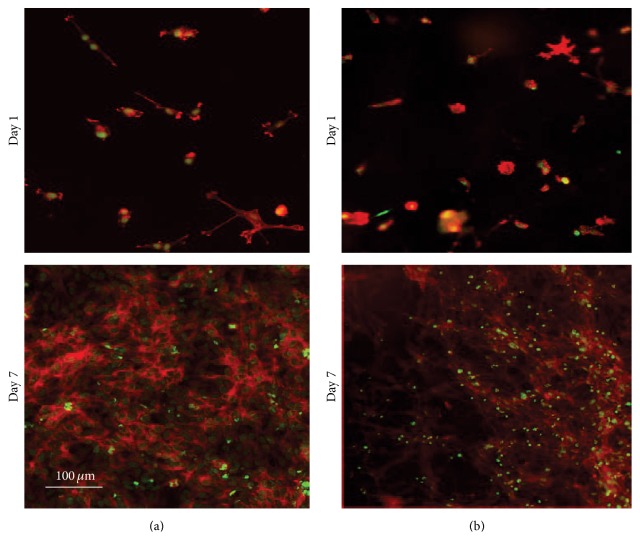
Fluorescence microscopy images of the NSC morphology on submicrofiber (b) in comparison to the TCPS (a), nuclear stain: SYTOX® Green nucleic acid stains, and actin filament stains: Phalloidin-TRITC.

According to cell morphology observation by fluorescence microscopy, cells were well spread on the mats (Figure 8). These results demonstrated that electrospun PLA fibers produced by TFA/DCM binary solvent are capable of supporting NSC viability and proliferation in an appropriate culture condition.

## 4. Conclusion

The use of TFA as a cosolvent overcomes the difficulties of PLA electrospinning with conventional solvents. The electrospinning of PLA, dissolved in a mixture of DCM/TFA, runs smoothly without any droplet coagulation or bead formation, due to the low surface tension and high conductivity of the binary-solvent system. Therefore, it becomes possible to easily produce a large quantity of electrospun mat. Moreover, this technique allows the altering of fiber diameter from few hundred nanometers to a few microns by only changing process parameters. Finally, it is possible to produce PLA electrospun fibers with different diameters through controlling polymer solution properties and process parameters. These mats could be useful in many applications including scaffolds for biomedical applications. Electrospun PLA mats supported NSC adhesion and proliferation; thus, they can be used in developing efficient approaches for NSC expansion in neural tissue engineering applications.

## Figures and Tables

**Table 1:** Properties of the solvents used in this study [29].

Solvents	Molecular structure	Molecular formula	Boiling point (°C)	Acidity (pKa)	Viscosity mPa s (at 20°C)	Surface tension (mN/m)	Solubility parameter (cal^1/2^ cm^−3/2^)	Dielectric constant	Electrical conductivity (*µ*s/cm^−1^)	Molar mass g mol^−1^
Chloroform	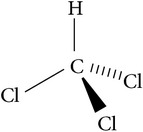	CHCl_3_	61	15.7 (20 °C)	0.56	27	9	4.8	1 × 10^−4^	119.38
DCM (dichloromethane)	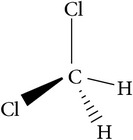	CH_2_Cl_2_	40		0.45	28	10	9	4 × 10^−5^	84.93
DMF (dimethylformamide)	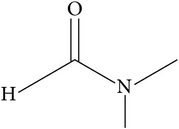	C_3_H_7_NO	153		0.92	35	12	37	6 × 10^−2^	73.09
TFA (trifluoroacetic acid)	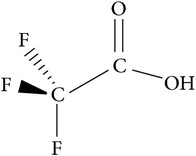	C_2_HF_3_O_2_	72	0.23	1.8	72.5				114.02

^*∗*^The *electric dipole moment* is a measure of the separation of positive and negative electrical charges in a system of electric charges, that is, a measure of the charge system's overall polarity.

Surface tension: TFA > DMF > DCM > CHLOROFORM.

Electric conductivity: TFA > DMF > CHLOROFORM > DCM.

Dielectric constant: TFA > DMF > DCM > CHLOROFORM.

Boiling point (°C): DMF > TFA > CHLOROFORM > DCM.

**Table 2:** Electrospinning conditions of PLA mats.

Sample: PLA- 1 g	Polymer solution			Process parameters				Collector					Ambient conditions	
	Solvent	Concentration (w/v%)^*∗*^	Age (day)	Needle size (G)	Tip-collector distance (cm)	Voltage (kV)	Flow rate (mL/h)	Dimension		Speed			RRH (%)	Temp. (°C)
								Length (cm)	Radius (m)	MD		TD (m/min)		
										Linear velocity (m/s)^*∗∗*^	Angular velocity (rpm)			
A1	TFA-DCM: (1 : 1)	7.7	0	22	15	16–18	0.5	—	0.075	2.25	300	0.3	10–15	22
A2										3.6	480	1		

B1	TFA-DCM: (1 : 1)	10	0	17	13.5	23–30	10			2.25	300	0.3	10–15	22–26
B2										3.6	480	1		

MD: machine direction; TD: transverse direction.

^*∗*^w/v (%) = mass solute (g) ÷ volume solution (mL) × 100.

^*∗∗*^The general equation: *V* (linear velocity) (m/s) = *r* (radius) (m) *∗* RPM (rounds/min) *∗* 0.1 (*w* or angular velocity) or 2*∗*3.14*∗R∗*RPM/60.

**Table 3:** Electrospun PLA mat properties.

Sample type	Collector speed in machine direction	Thickness (*µ*m) ± SD	Porosity% ± SD	Sample average fiber diameter ± SD^*∗*^
A1: 7.7% (w/v)	2.25	58 ± 3	87 ± 1	600 ± 100 nm
A2: 7.7% (w/v)	3.6	53 ± 1	86 ± 2	500 ± 50 nm
B1: 10% (w/v)	2.25	62 ± 6	84 ± 0.5	3 ± 0.2 *µ*m
B2: 10% (w/v)	3.6	58 ± 2	83 ± 1	2 ± 0.2 *µ*m

^*∗*^ *p* < 0.05.

**Table 4:** PLA solution properties of different concentrations in single and binary-solvent systems.

Polymer solution concentration	Solvent type	Viscosity (mpa s)	Surface tension (mN/m)	Conductivity (*µ*S cm^−1^)
3%	DCM	5.3 ± 0.1	27.4 ± 0.3	negligible
7%		26.5 ± 0.2	30.2 ± 1.5	

3%	Chloroform	11.8 ± 0.4	26.7 ± 0.6	negligible
7%		86 ± 0.7	27.7 ± 0.3	

3%	DCM/chloroform	6.3 ± 0.1	28.2 ± 0.4	negligible
7%		44.8 ± 0.1	28.9 ± 0.2	

7.7%	DCM/TFA	52.9 ± 0.1	19.6 ± 0.5	2.3 ± 0.2
10%	DCM/TFA	77.2 ± 0.2	19.9 ± 0.5	
